# Phenolics Content and Antioxidant Activity of Tartary Buckwheat from Different Locations

**DOI:** 10.3390/molecules16129850

**Published:** 2011-11-25

**Authors:** Xu-Dan Guo, Yu-Jie Ma, John Parry, Jin-Ming Gao, Liang-Li Yu, Min Wang

**Affiliations:** 1 College of Food Science and Engineering, Northwest A & F University, YangLing, Shaanxi 712100, China; Email: guoxudan123@126.com (X.-D.G.); 2 Agricultural Research Station, Virginia State University, Petersburg, VA 23806, USA; 3 College of Sciences, Northwest A & F University, YangLing, Shaanxi 712100, China; 4 Department of Nutrition and Food Science, University of Maryland, College Park, MD 20742, USA

**Keywords:** antioxidant activity, environment, phenolics, flavonoids, phenolic acid, tartary buckwheat, variety

## Abstract

Two tartary buckwheat samples (Xingku No.2 and Diqing) grown at three locations were analyzed for free and bound phenolic content and antioxidant properties. Moreover, the relative contributions of variety and growing environment to phenolic content and antioxidant properties were determined, as well as correlations of these properties to growing conditions. The total phenolic contents varied from 5,150 to 9,660 μmol of gallic acid equivalents per 100 gram of dry weight (DW) of tartary buckwheat and the free phenolics accounted for 94% to 99%. Rutin content was in the range from 518.54 to 1,447.87 mg per 100 gram of DW of tartary buckwheat. *p*-Hydroxybenzoic, ferulic and protocatechuic acids were the prominent phenolic acids and other phenolics, including *p*-coumaric, gallic, caffeic, vanillic and syringic acids were also detected. Tartary buckwheat exhibited higher DPPH^●^ and ABTS^●+^ scavenging activities and was more effective at preventing the bleaching of β-carotene in comparison with reference antioxidant and plant phenolics constituents. Additionally, growing conditions and the interaction between variety and environment may have more contribution than variety to individual phenolics and antioxidant properties of tartary buckwheat. Environmental parameters such as higher altitudes may also have an increasing effect on rutin and phenolic acids. This study suggests that tartary buckwheat has potential health benefits because of its high phenolic content and antioxidant properties. These components could also be enhanced by optimizing the growing conditions of a selected variety.

## 1. Introduction

Tartary buckwheat (*Fagopyrum tartaricum* Gaertn.) belongs to the Polygonaceae family, which is one of the two cultivated varieties, along with common buckwheat. Tartary buckwheat is grown and used in the mountainous regions of southwest China, northern India, Bhutan and Nepal. Recently, as a unique food medicine dual-use cereal crop, tartary buckwheat has become more popular among consumers for both its nutritional and medicinal values. Epidemiological studies have revealed that buckwheat can reduce the risk of chronic diseases [1]. The beneficial effects of buckwheat have been attributed to its high levels of polyphenol compounds such as flavonoids, which exhibit antioxidant activity [2]. Structurally, the polyphenols comprise several hundred molecules (*i.e.*, benzene rings with one or more hydroxyl groups) in edible plants and can be very generally divided to flavonoids and phenolic acids [3,4]. Though termed secondary metabolites, polyphenols play a vital part in the protection of plant against UV radiation, pathogens and herbivores, and help maintain structural integrity for the cell wall [5,6]. Most naturally-occurring polyphenols in edible plants are found either free or bound mainly by ester and ether linkages to polysaccharides and proteins [7,8]. The dissolution of polyphenols varies in the different regions of the gastrointestinal tract due to their structures [9]. However, little information is known about the comparison of the antioxidant properties of free and bound phenolics in tartary buckwheat. Therefore, it is essential to establish a complete profile of polyphenols within the tartary buckwheat seed prior to studying the effect of tartary buckwheat seed on reducing the incidence of human diseases.

It is well known that plant polyphenol content and antioxidant property depend on a number of factors such as variety, location and environmental conditions [8,10]. Studies have showed that antioxidant activities were influenced by various environmental parameters including total solar radiation, temperature stress, water stress and light intensity for wheat [11,12], strawberry [13] and *Hypericum brasiliense* [14]. Previous studies of buckwheat antioxidants also suggested that location, environmental factors, growing season and cultivar influenced the phenolics, flavonoids and rutin content of common buckwheat seed or hull [15,16,17]. A new report found that altitudinal variations have profound effects on the polyphenol content and antioxidant activity of tartary buckwheat of western Himalaya [18]. Few comprehensive studies, however, have separated the effects of variety and environment and quantified their contributions to individual antioxidant activity variances for tartary buckwheat. Therefore, evaluation of how variety and growing conditions affect phenolics levels and antioxidant properties of tartary buckwheat is important step to optimize the growing conditions of a selected variety to produce tartary buckwheat rich in natural antioxidants for disease prevention and health promotion.

Emphasis on the distribution of free and bound phenolics and antioxidant activity is critical to understand the potential health benefits of tartary buckwheat. It is also useful to quantify the contributions of variety (V), growing environment (E) and their interaction (V × E) to antioxidant activity variance of tartary buckwheat for breeders and growers. The objectives of the present study were the following: (i) to determinate the total phenolic, flavonoid content and phenolic acid composition of free and bound phenolics; (ii) to measure the antioxidant activities of free and bound phenolics; (iii) to elucidate the effects of V, E and V × E on the antioxidant activities of tartary buckwheat by quantifying their separate contributions to antioxidant activity variance and investigate the effects of environmental parameters including mean temperature, amount of precipitation, sunlight hours and altitude on the antioxidant properties of tartary buckwheat. 

## 2. Results and Discussion

### 2.1. Total Phenolics and Flavonoids Content

The phenolic content in the tartary buckwheat was examined first (Table 1). The free phenolic content (4,820–9,590 μmol of gallic acid eq./100 g DW) was 93–98-fold higher (*P* < 0.05) than the respective bound phenolic content (71–394 μmol of gallic acid eq./100 g DW) in the six samples tested, showing that most phenolics in tartary buckwheat were present in the free form. Meanwhile, the free phenolic content of tartary buckwheat was higher than that of corn (23-45-fold), wheat (25-50-fold) [9], cranberry (2-13-fold) and apple (3-6-fold) [7]. In our study, the bound phenolic content of Diqing tartary buckwheat grown at Sichuan (394 ± 3 μmol of gallic acid eq./100 g dried grain) was the highest among the six samples and slightly higher than that of rice (346 ±13 μmol of gallic acid eq./100 g DW) [9], indicating that the bound phenolics in tartarty buckwheat should not be neglected. The total phenolic content of the tested buckwheat flour ranged from 5,150 to 9,660 μmol of gallic acid eq./100 g DW, which suggests that the levels of phenolics in tartary buckwheat are higher than those found in some fruits, vegetables and other cereals when expressed on a per 100 g dry weight basis. For example, the total phenolic content of tartary buckwheat was much higher than that of cranberry, apple [7], raspberry [19], honey [20], corn, wheat, oats and rice [9], suggesting that tartary buckwheat may serve as an excellent dietary source of phenolics. In this study, tartary buckwheat, Xingku No.2 from Sichuan had the highest phenolic content, followed by Diqing from Gansu. Interestingly, the phenolic content of Qiqing variety from Gansu was almost two times higher than that of the Xingku No.2 variety from Gansu. These results may be due to the interaction among the environmental conditions. Because low temperature may increase production of phenolics by enhancing synthesis of phenylalanine ammonia lyase (PAL) in plants, while high altitude and long sunlight hours with higher UV radiation positively affect the activity of phenolics synthase [18]. Meanwhile, small amounts of precipitation could enhance the defense system of plant against stress, leading to an increased phenolic content [21].

The free phenolic content of each tested sample was significantly higher than bound phenolics (*P* < 0.05). Table 1 clearly showed that most tartary buckwheat phenolics were in the free fraction, which was consistent with the results reported by Hung and Morita [22], who found that common buckwheat phenolics existed predominantly in the free form. By contrast, the phenolics in wheat, rye, corn, oat and rice exist primarily in the bound form [9,23]. Research suggests that free phenolics may be digested in the upper gastrointestinal tract, while bound phenolics may reach the colon and exert their health benefits [9]. Therefore, phenolics in tartary buckwheat may be more readily available in the upper gastrointestinal tract compared to wheat, corn, rice and oat. Therefore, the unique phenolics in tartary buckwheat complement those in wheat, corn, rice and oat when consumed together. Moreover, bound phenolic compounds of tartary buckwheat tested cannot be ignored as their content was much higher than that of rice that contained more bound phenolics than free phenolics.

In the tartary buckwheat samples tested, the free flavonoid contents were higher than the bound flavonoid contents (Table 1). This was different from the flavonoid distribution in common buckwheat [22], wheat, rice, corn and oat [9], whose bound flavonoid contents was higher than the free flavonoids. The free flavonoids ranged from 76% in Diqing from Sichuan and Ningxia to 95% in Xingku No.2 from Ningxia (Table 1). The free flavonoid content of Xingku No.2 from Ningxia was significantly higher than those of the other tested samples (*P* < 0.05) (Table 1); therefore, it may play more preventive and protective role in upper gastrointestinal tract compared to other samples. The bound flavonoid contents of Diqing from Ningxia and Sichuan were similar and both were significantly higher than that of other samples (*P* < 0.05) (Table 1), and they may be more beneficial in delivering bound flavonoids to the colon compared to other samples. Total flavonoid contents ranged from 2,077–3,149 μmol of rutin eq./100 g DW, which were much higher than that of common buckwheat [15] and tartary buckwheat in western Himalaya [18]. Total flavonoid contents followed a similar pattern as free flavonoid contents in all tested samples, because of the large contribution from free flavonoids. Flavonoids are important phytochemical components of tartary buckwheat and they have potent antioxidant and anticancer activity [9].

**Table 1:** Phenolic and flavonoid content of tartary buckwheat.

Variety	Location	Phenolic content			Flavonoid content		
		(μmol of gallic acid eq./100 g DW)			(μmol of rutin eq./100 g DW)		
		Free	Bound	Total	Free	Bound	Total
Xingku No.2	Sichuan	9590 ± 428 a	71 ± 10 d	9660 ± 433 a	1980 ± 210 bc	97 ± 12 d	2077 ± 198 c
	Ningxia	8410 ± 621 b	353 ± 16 ab	8760 ± 614 abc	3014 ± 188 a	135 ± 23 d	3149 ± 187 a
	Gansu	4820 ± 260 d	333 ± 26 b	5150 ± 283 d	2161 ± 170 b	318 ± 14 c	2479 ± 157 bc
Diqing	Sichuan	7310 ± 412 c	394 ± 3 a	7700 ± 414 c	1719 ± 77 c	541 ± 5 a	2260 ± 81 bc
	Ningxia	8150 ± 337 bc	253 ± 13 c	8400 ± 342 bc	1871 ± 124 bc	593 ± 85 a	2464 ± 151 bc
	Gansu	8950 ± 138 ab	310 ± 20 b	9260 ± 118 ab	2109 ± 84 bc	425 ± 26 b	2534 ± 102 b

Data are presented as mean ± SD (*n* = 3). Within each column, means with the same letter are not significantly different (*P* < 0.05).

### 2.2. Phenolic Compound Profiles

Both phenolic acids and flavonoids, which are phenolics, are natural products commonly found in many cereal grains. Ferulic, vanillic and syringic acids were found as the major phenolic acids in wheat [24] and flavonoids were also found in wheat, rice, corn and oat [9]. Common buckwheat was found to contain rutin, phenolic acids and tocopherols [15,25]. In this study, the phenolic acids such as *p*-hydroxybenzoic, ferulic, protocatechuic, *p*-coumaric, gallic, vanillic, caffeic and syringic acids and the flavonoids such as rutin, quercetin and catechin were detected in the free and bound phenolic extracts of tartary buckwheat using a high-performance liquid chromatography (HPLC) system (Table 2). As shown in Table 2, *p*-hydroxybenzoic, ferulic, protocatechuic acid were the prominent phenolic acids in tartary buckwheat, which accounted for 83–88% of total phenolic acid, and the concentration of rutin was the highest among the three flavonoid compounds, followed by quercetin, and catechin was the lowest. The total concentrations of phenolic acids and flavonoids in the free phenolic extracts were significantly higher than that in the bound phenolic extracts in each tartary buckwheat sample (*P* < 0.05). These results were consistent with the results of total phenolic content determined by the Folin-Ciocalteu method. Among the eight phenolic acids, *p*-hydroxybenzoic, ferulic, protocatechuic, *p*-coumaric, gallic and vanillic acids were present in all six tartary buckwheat samples, whereas caffeic and syringic acids were detected in individual samples (Table 2), which differs from the phenolic acid composition of common buckwheat because protocatechuic acid was not detected in the latter [5,22,26]. Variety and growing location influenced the phenolic acid and flavonoid concentration in this study. The total phenolic acid and flavonoid content of Xingku No.2 were higher than those of Diqing (Table 2). The total phenolic acid content of Xingku No.2 and Diqing were 10.66–19.91 mg/100g DW and 8.91–14.99 mg/100g DW, respectively, and the flavonoid content of Xingku No.2 and Diqing were 1,653–1,991 mg/100g DW and 1,385–1,897 mg/100g DW, respectively.

**Table 2:** Flavonoid and phenolic acid composition in tartary buckwheat seed.

Variety	Location	Free (mg/100 g DW)	Bound (mg/100 g DW)	Total (mg/100 g DW)
(A) Rutin composition				
Xingku No.2	Sichuan	1444.59 ± 1.75 a	3.28 ± 0.06 d	1447.87 ± 1.69 a
	Ningxia	1213.98 ± 9.05 e	2.94 ± 0.04 e	1216.92 ± 9.09 e
	Gansu	1344.47 ± 5.86 b	3.83 ± 0.04 b	1348.30 ± 5.90 b
Diqing	Sichuan	1322.00 ± 10.59 c	3.59 ± 0.06 c	1325.59 ± 10.65 c
	Ningxia	517.45 ± 4.34 f	1.09 ± 0.03 f	518.54 ± 4.32 f
	Gansu	1247.01 ± 6.74 d	11.49 ± 0.04 a	1258.50 ± 6.77 d
(B) Quercetin composition				
Xingku No.2	Sichuan	478.76 ± 2.39 d	0.61 ± 0.01 c	479.37 ± 2.40 d
	Ningxia	425.09 ± 4.03 e	0.56 ± 0.01 d	425.65 ± 4.03 e
	Gansu	621.82 ± 2.28 b	0.72 ± 0.02 b	622.54 ± 2.29 b
Diqing	Sichuan	538.42 ± 2.60 c	0.61 ± 0.02 c	539.03 ± 2.61 c
	Ningxia	857.23 ± 3.66 a	0.39 ± 0.01 e	857.62 ± 3.66 a
	Gansu	626.59 ± 3.14 b	0.86 ± 0.03 a	627.46 ± 3.12 b
(C) Catechin composition				
Xingku No.2	Sichuan	4.40 ± 0.03 a	7.61 ± 0.03 d	12.01 ± 0.05 b
	Ningxia	3.74 ± 0.03 b	6.34 ± 0.05 e	10.08 ± 0.05 d
	Gansu	3.13 ± 0.04 c	16.84 ± 0.04 a	19.96 ± 0.01 a
Diqing	Sichuan	0.95 ± 0.02 f	8.83 ± 0.04 c	9.78 ± 0.06 e
	Ningxia	2.95 ± 0.03 d	5.94 ± 0.04 f	8.89 ± 0.06 f
	Gansu	2.31 ± 0.04 e	9.02 ± 0.07 b	11.34 ± 0.06 c
(D) *p*-Hydroxybenzoic acid composition				
Xingku No.2	Sichuan	5.39 ± 0.15 b	0.11 ± 0.01 d	5.51 ± 0.14 b
	Ningxia	2.95 ± 0.03 c	0.14 ± 0.01 c	3.10 ± 0.03 c
	Gansu	8.56 ± 0.32 a	0.21 ± 0.01 a	8.78 ± 0.31 a
Diqing	Sichuan	5.64 ± 0.07 b	0.10 ± 0.01 e	5.74 ± 0.07 b
	Ningxia	2.22 ± 0.00 c	nd	2.22 ± 0.00 c
	Gansu	5.00 ± 0.03 b	0.19 ± 0.01 b	5.18 ± 0.04 b
(E) Ferulic acid composition				
Xingku No.2	Sichuan	6.4 ± 0.42 a	0.89 ± 0.01 b	7.29 ± 0.39 a
	Ningxia	2.07 ± 0.01 e	0.78 ± 0.01 d	2.85 ± 0.01 e
	Gansu	1.00 ± 0.01 f	0.86 ± 0.00 c	1.86 ± 0.01 f
Diqing	Sichuan	4.31 ± 0.09 b	0.61 ± 0.01 e	4.92 ± 0.07 b
	Ningxia	2.73 ± 0.21 d	0.48 ± 0.02 f	3.21 ± 0.20 d
	Gansu	3.73 ± 0.12 c	0.98 ± 0.04 a	4.71 ± 0.11 c
(F) Protocatechuic acid composition				
Xingku No.2	Sichuan	3.16 ± 0.11 a	1.47 ± 0.01 b	4.63 ± 0.13 a
	Ningxia	1.81 ± 0.02 b	1.41 ± 0.02 c	3.21 ± 0.02 c
	Gansu	1.58 ± 0.03 c	2.15 ± 0.02 a	3.73 ± 0.04 b
Diqing	Sichuan	1.32 ± 0.05 d	0.4 ± 0.00 e	1.73 ± 0.06 f
	Ningxia	1.6 ± 0.01 c	0.5 ± 0.03 d	2.1 ± 0.04 e
	Gansu	1.14 ± 0.01 e	1.49 ± 0.04 b	2.64 ± 0.05 d
(G) *p*-Coumaric acid composition				
Xingku No.2	Sichuan	0.72 ± 0.03 a	0.26 ± 0.01 a	0.98 ± 0.04 a
	Ningxia	0.23 ± 0.00 d	nd	0.23 ± 0.00 e
	Gansu	0.5 ± 0.02 b	0.18 ± 0.02 b	0.68 ± 0.03
Diqing	Sichuan	0.38 ± 0.01 c	0.11 ± 0.00 c	0.49 ± 0.01 c
	Ningxia	0.18 ± 0.02 e	0.11 ± 0.01 c	0.29 ± 0.02 d
	Gansu	0.51 ± 0.01 b	nd	0.51 ± 0.01 c
(H) Gallic acid composition				
Xingku No.2	Sichuan	0.62 ± 0.01 a	nd	0.62 ± 0.01 a
	Ningxia	0.48 ± 0.01 c	nd	0.48 ± 0.01 c
	Gansu	0.48 ± 0.00 c	nd	0.48 ± 0.00 c
Diqing	Sichuan	0.48 ± 0.00 c	nd	0.48 ± 0.00 c
	Ningxia	0.49 ± 0.02 c	nd	0.49 ± 0.02 c
	Gansu	0.55 ± 0.05 b	nd	0.55 ± 0.05 b
(I) Caffeic acid composition				
Xingku No.2	Sichuan	0.49 ± 0.00 a	nd	0.49 ± 0.00 a
	Ningxia	nd	0.12 ± 0.00 b	0.12 ± 0.00 b
	Gansu	0.23 ± 0.01 c	0.12 ± 0.00 b	0.35 ± 0.00 c
Diqing	Sichuan	nd	nd	nd
	Ningxia	0.32 ± 0.02 b	nd	0.32 ± 0.02 d
	Gansu	0.19 ± 0.01 d	0.17 ± 0.02 a	0.36 ± 0.03 b
(J) Vanillic acid composition				
Xingku No.2	Sichuan	nd	0.21 ± 0.01 d	0.21 ± 0.01 f
	Ningxia	0.53 ± 0.01c	0.14 ± 0.01 e	0.67 ± 0.02 c
	Gansu	nd	0.52 ± 0.00 a	0.52 ± 0.00 d
	Sichuan	1.17 ± 0.01a	0.43 ± 0.01 b	1.6 ± 0.01 a
	Ningxia	nd	0.28 ± 0.02 c	0.28 ± 0.02 c
	Gansu	0.6 ± 0.00b	0.43 ± 0.01 b	1.04 ± 0.01 b
(K) Syringic acid composition				
Xingku No.2	Sichuan	0.18 ± 0.01 a	nd	0.18 ± 0.01 a
	Ningxia	nd	nd	nd
	Gansu	0.12 ± 0.00 b	nd	0.12 ± 0.00 b
Diqing	Sichuan	nd	nd	nd
	Ningxia	nd	nd	nd
	Gansu	nd	nd	nd

Values are mean ± SD (*n* = 3). Values with the same letter in common within each column are significantly different (*P* < 0.05). nd, not detected.

Comparing the three growing locations, the total phenolic acid content of both Xingku No.2 and Diqing from Sichuan were significantly higher than those from Gansu and Ningxia, while the flavonoid content of Xingku No.2 and Diqing from Gansu were significantly higher than those from Sichuan and Ningxia (*P* < 0.05).

### 2.3. Antioxidant Properties

**Figure 1:**
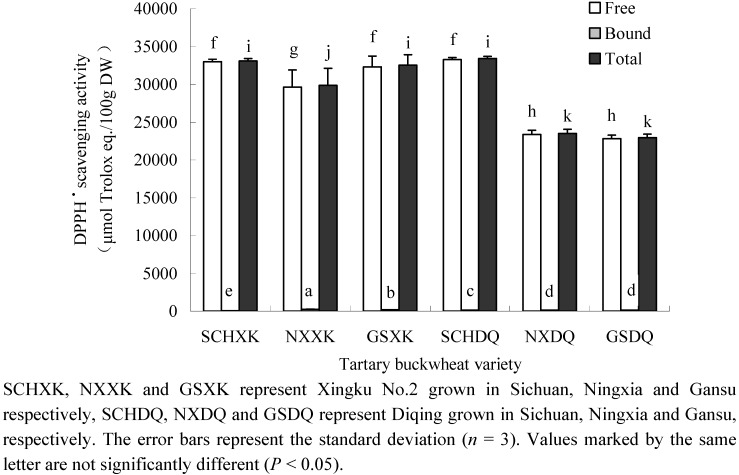
DPPH radical scavenging activity of two tartary buckwheat varieties grown at 3 locations (μmol Trolox eq /100 g DW).

**Figure 2:**
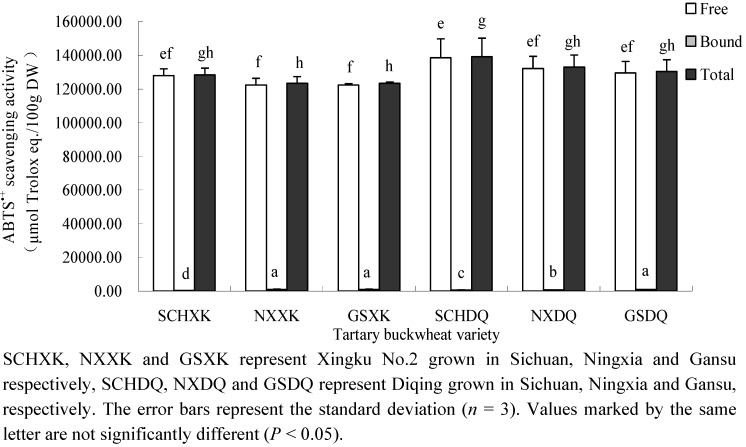
ABTS^●+^ scavenging activity of two tartary buckwheat varieties grown at 3 locations (μmol Trolox eq /100g DW).

**Figure 3:**
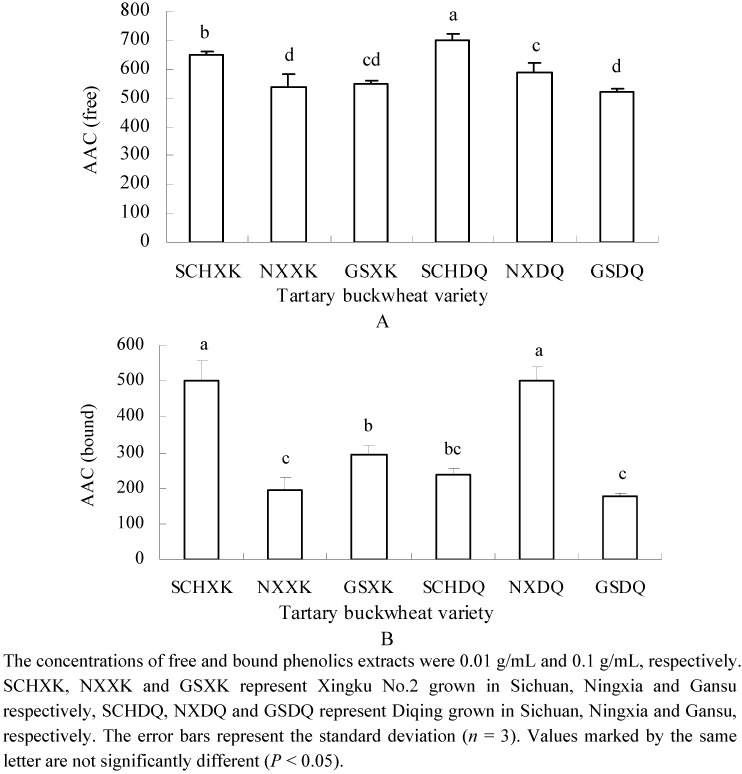
Antioxidant activity coefficient (AAC) of free (A) and bound (B) phenolics of two tartary buckwheat varieties grown at three locations.

Tartary buckwheat showed significant DPPH^●^ and ABTS^●^^+^ scavenging activities and effectiveness in preventing the bleaching of β-carotene in a β-carotene-linoleate model system (Figure 1,Figure 2,Figure 3). As can be seen, the radical scavenging capacity of free phenolic compounds was greater than that of the bound phenolic compounds. On average, free phenolics contributed more than 99% of the total radical scavenging capacity. Therefore, the free phenolic extracts were considered to be the major contributors of the total radical scavenging capacity. In this study, DPPH^●^ and ABTS^●+^ scavenging activity was in the range from 2.3 × 10^4^ to 3.3 × 10^4^ μmol Trolox eq./100g DW (Figure 1) and 1.2 × 10^5^ to 1.4 × 10^5^ μmol Trolox eq./100 g DW (Figure 2), respectively. The free radical scavenging capacity of tartary buckwheat was greater than that of wheat [12] and bio-fortified carrots [27], suggesting that tartary buckwheat may serve as excellent dietary source of free radical scavengers. In terms of effectiveness at preventing the bleaching of β-carotene, the antioxidant activity coefficient (AAC) of the free phenolics was higher than that of the bound phenolics in the tartary buckwheat, and the AAC of free and bound phenolics ranged from 518 to 701 (Figure 3A) and from 178 to 501 (Figure 3B), respectively. In a previous report, the AACs of common buckwheat seed, wheat germ, sunflower seed and blueberry were 125, 236, 298 and 796, respectively [28]. These data indicated that the AAC of free phenolics of tartary buckwheat was comparable to, or even higher than certain fruits and grains, although the concentration of free phenolics in this study was lower than that of fruits and grains. The results also suggest that tartary buckwheat has a higher comparative effectiveness at preventing the bleaching of β-carotene in the β-carotene-linoleate model system.

### 2.4. Effects of V and E on Tartary Buckwheat Phenolics Content and Antioxidant Properties

The above results support the assumption that variety and environment may have remarkable effects on the phenolics and antioxidant properties of tartary buckwheat. To separate and quantify the contribution of V, E and V × E interactions on tartary buckwheat antioxidant property and phenolics content variance, a 2 × 3 factorial designed ANOVA was conducted on the data from the two tartary buckwheat varieties grown in three locations. The magnitude of variance proportion (percent total mean squares) attributed to V, E and V × E indicates their relative significance in determining each antioxidant property. Results showed that V, E and V×E significantly influenced antioxidant properties of tartary buckwheat except the ABTS^●+^ scavenging activity, AAC and TFC (*P* < 0.05) (Table 3). As for AAC, E contributed the highest proportion (77%) of total variance for free phenolics and V × E contributed the highest proportion (77%) of total variance for bound phenolics. In terms of TFC, E and V × E contributed 52% and 27% of total variance, respectively. For the antioxidant activity, E contributed the highest proportion of total variance, ranging from 40 to 77%, whereas V contributed 31–33% and V × sE contributed 20–77%. For the phenolics data, E contributed the highest proportion of total variance, ranging from 6 to 79%, V and V × E ranged from 3.5 to 75% and 5.8 to 71%, respectively. Although revealing significant information for determining the separate effects of V and E on antioxidant properties, the scope of these results was limited by the small number of samples involved. The contributions of V, E and V × E on antioxidant properties of several more tartary buckwheat varieties should be investigated to increase the scope of our results in further study.

**Table 3:** Proportions of variance attributed to variety (V), environment (E) and V × E interaction for 2 tartary buckwheat varieties grown in 3 locations ^a^.

Antioxidant property	Variance component		
	V	E	V × E
DPPH^●^ scavenging activity (μmol Trolox eq./100 g DW)	33.25 **	40.23 **	20.37 **
ABTS^●+^ scavenging activity (μmol Trolox eq./100 g DW)	31.47 *	7.42	n
AAC (free phenolics)	2.45	77.36 **	6.33 *
AAC (bound phenolics)	0.41	16.23 **	77.11 **
Total Phenolic Content (μmol of gallic acid eq./100 g DW)	3.48 *	18.89 **	71.12 **
Total Flavonoid Content (μmol of rutin eq./100 g DW)	3.40	52.01 **	27.14 **
Total phenolic acid (mg/100g DW)	18.86 **	66.45 **	14.68 **
Rutin (mg/100 g DW)	6.96 **	46.86 **	46.13 **
Quercetin (mg/100 g DW)	19.40 **	72.87 **	7.69 **
Catechin (mg/100 g DW)	29.47 **	50.79 **	19.72 **
*p*-Hydroxybenzoic acid (mg/100 g DW)	5.24 **	79.19 **	5.75 *
Ferulic acid (mg/100 g DW)	4.08 **	55.83 **	40.08 **
Protocatechuic acid (mg/100 g DW)	75.67 **	5.97 **	18.28 **

^a^ Results expressed as percent of total variation (mean squares) from factorial design ANOVA using variety and environment as fixed effects. Results with asterisks were significant at *P* < 0.05; *, significant (*P* < 0.05); **, highly significant (*P* < 0.01). AAC, antioxidant activity coefficient. n, no contribution

Growing environment (E) may be a significant factor affecting some antioxidant properties for tartary buckwheat flour. The effects of environmental parameters including mean temperature, amount of precipitation, sunlight hours and altitude on the antioxidant properties of tartary buckwheat were investigated. Correlation analysis found significant positive correlations between altitude and rutin or total phenolic acid content for both Xingku No.2 and Diqing (*P* < 0.05). No significant correlation among the other three environmental parameters and antioxidant property was detected for the two varieties tested (*P* < 0.05) (Table 4). The effects of altitude on polyphenol and antioxidant property of tartary buckwheat have been reported in a recent study [18]. Our results support the notion that higher altitude, which is often linked to higher UV radiation, causes an increase in rutin content.

**Table 4:** Correlation analysis of growing conditions and antioxidant properties and phenolics for two tartary buckwheat varieties grown in 3 locations.

Environment	Variety	Antioxidant property and phenolic content										
parameter		DPPH	AAC_F_	AAC_B_	TPC	TFC	TPA	R	Q	HA	FA	PA
MT (°C)	XK	−0.72	−0.22	−0.26	0.80	0.56	−0.56	−0.50	−0.98 *	−0.99 *	0.25	−0.29
	DQ	0.13	0.46	0.91	−0.61	−0.32	−0.83	−0.78	0.64	−0.73	−0.76	−0.65
AOP (mm)	XK	0.85	0.98 *	0.99 *	0.38	−0.94	0.94	0.96 *	0.06	0.23	0.88	0.99 *
	DQ	0.93	0.75	−0.63	−0.62	−0.84	0.74	0.79	−0.90	0.84	0.82	−0.58
SH (h)	XK	−0.98 *	−0.57	−0.75	0.34	0.92	−0.93	−0.90	−0.72	−0.83	−0.33	−0.77
	DQ	−0.44	−0.11	0.99 *	−0.07	0.25	−0.99 *	−0.99 *	0.96 *	−0.99 *	−0.99 *	−0.12
A (m)	XK	0.99 *	0.77	0.9	−0.07	−0.99 *	0.99 *	0.98 *	0.51	0.64	0.57	0.91
	DQ	0.67	0.37	−0.91	−0.2	−0.51	0.97 *	0.98 *	−0.99 *	0.99 *	0.99 *	−0.15

Results expressed as Pearson correlation coefficients with indicated level of significance. Data with asterisks were significant at *P* < 0.05. XK, Xingku No.2; DQ, Diqing; MT, mean temperature; AOP, amount of precipitation; SH, sunlight hours; A, altitude; DPPH, DPPH^●^ scavenging activity; AAC_F_, antioxidant activity coefficient of free phenolics; AAC_B_, antioxidant activity coefficient of bound phenolics; TPC, total phenolic content; TFC, total flavonoid content; TPA, total phenolic acid ; R, rutin; Q, quercetin; HA, *p*-Hydroxybenzoic acid; FA, Ferulic acid; PA, Protocatechuic acid.

### 2.5. Correlations Between Antioxidant Properties

Possible correlations between the antioxidant properties of tartary buckwheat samples were observed (Table 5). No significant correlation was found between antioxidant property and phenolic content (*P* < 0.05), indicating that phenolics were not the only components responsible for the antioxidant property of tartary buckwheat, obviously, other factors should be involved and the possibilities deserve further investigation. However, two significant correlations were found between rutin content and quercetin content (*r* = –0.83, *P* < 0.05) or total phenolic acid content (*r* = 0.81, *P* < 0.05). The significant negative correlation between rutin and quercetin content is consistent with the fact that quercetin is a phenolic glycoside of rutin. The significant positive correlation between rutin and total phenolic acid content showed that tartary buckwheat containing higher rutin might contain higher phenolic acid. Further research using a larger sample size is needed to retest the correlation between the rutin and phenolic acid content of tartary buckwheat.

**Table 5:** Significant correlations between antioxidant properties for 6 tartary buckwheat samples.

	DPPH	ABTS	AAC_F_	AAC_B_	TPC	TFC	TPA	R	Q
DPPH	1								
ABTS	−0.09	1							
AAC_F_	0.56	0.71	1						
AAC_B_	0.01	0.1	0.37	1					
TPC	−0.36	0.22	0.16	0.16	1				
TFC	−0.26	−0.53	−0.56	−0.56	−0.01	1			
TPA	0.58	−0.07	0.33	0.1	−0.04	−0.65	1		
R	0.68	−0.23	0.15	−0.42	−0.08	−0.16	0.81 *	1	
Q	−0.66	0.3	−0.14	0.43	−0.19	−0.21	−0.46	−0.83 *	1

Results expressed as Pearson correlation coefficients with indicated level of significance. Data with asterisks were significant at *P* < 0.05. DPPH, DPPH^●^ scavenging activity; ABTS, ABTS^●+^ scavenging activity; AAC_F_, antioxidant activity coefficient of free phenolics; AAC_B_, antioxidant activity coefficient of bound phenolics; TPC, total phenolic content; TFC, total flavonoid content; TPA, total phenolic acid; R, rutin; Q, quercetin.

## 3. Experimental

### 3.1. Tartary Buckwheat Sample Preparation

The two commercial varieties of tartary buckwheat were Xingku No.2 and Diqing. They were planted in three testing locations of significantly different altitudes: Liangshan in Sichuan (SCH), Tongxin in Ningxia (NX) and Dingxi in Gansu (GS) in China. Seeds were sown 3 cm apart and the rows were separated by 33 cm by randomizing block arrangement in the experimental field of 10 m^2^. The tartary buckwheat grown in Sichuan, Ningxia and Gansu were sown on April 14, July 8 and May 30, 2009, respectively. The main difference in growing conditions tested including mean temperature, amount of precipitation, sunlight hours and altitude during the whole growing period of tartary buckwheat are presented in Table 6. After harvest, the grains were dried at room temperature and debris was removed. All samples were ground by a FW100-High Speed Universal Grinder (China) to pass a 40 mesh sieve.

**Table 6:** Environment parameters during the whole growing period at 3 tartary buckwheat growing locations in China.

Location	Mean	Amount	Sunlight hours	Altitude
	temperature (°C)	of precipitation (mm)	(h)	(m)
Sichuan	17.19	667.3	1078.4	2100
Ningxia	19.61	151.4	1446.5	1422
Gansu	14.02	317	1071.7	1920

### 3.2. Chemicals

The 1,1-diphenyl-2-picrylhydrazyl radical (DPPH^●^), 2,2′-azinobis-(3-ethylbenzthiazoline-6-sulfonate) (ABTS), 6-hydroxy-2,5,7,8-tetramethylchroman-2-carboxylic acid (Trolox), Folin-Ciocalteu reagent and β-carotene were purchased from Sigma (Germany), while Tween-40 was purchased from Merck (Germany). Rutin, quercetin, catechin, *p*-hydroxybenzoic, ferulic, protocatechuic, *p*-coumaric, gallic, caffeic, vanillic and syringic acids were from Tianjin YiFang S & T (China). All other chemicals and solvents were of the highest commercial grade and used without further purification.

### 3.3. Extraction of Free and Bound Phenolic Compounds

The free and bound phenolic compounds were extracted by the method reported previously with slight modification [9,29]. One gram of flour powder was extracted with 80% chilled aqueous acetone (50 mL) for 10 min using a homogenizer. After centrifugation at 402 g for 10 min, the supernatant was collected. The extraction was repeated three times. Supernatants were combined, then vacuum-evaporated to dryness at 45 °C and finally reconstituted with methanol to a volume of 10 mL. The extracts were stored at −20 °C until use.

The residues from the extraction of free phenolic compound were then digested with 2 mol/L NaOH (20 mL) for 1 h with shaking under nitrogen gas at room temperature. The mixture was neutralized with concentrated hydrochloric acid (4 mL) and extracted with hexane (20 mL) to remove lipids. The final solution was extracted five times with ethyl acetate (20 mL) and the ethyl acetate fractions were collected, then vacuum-evaporated to dryness at 45 °C and finally reconstituted with methanol to a volume of 10 mL. The extracts were stored at −20 ° Cuntil use.

### 3.4. Determination of Total Phenolic Content

Total phenolic content of each extract was determined using the method described by Adom *et al.* with some modifications [11]. Briefly, extracts or control (125 μL) were mixed with distilled deionized water (500 μL) followed by addition of Folin-Ciocalteu reagent (125 μL) and then allowed to stand at room temperature for 6 min. Next, 7% aqueous sodium carbonate (1.25 mL) was added, followed by adjustment of the volume to 3 mL with deionized water. The absorbance was measured at 760 nm using a spectrophotometer (UV1240, Shimadzu, Japan) after reacting for 90 min in the dark. The total phenolic content of the tartary buckwheat sample was expressed as μmol of gallic acid eq./100 g DW (dry weight). The concentration range of gallic acid was 0–44 μmol/L.

### 3.5. Determination of Total Flavonoid Content

Total flavonoid content was determined by a colorimetric method described previously and modified in our laboratory [11]. Aliquots of extracts were reacted with 5% sodium nitrite (200 μL). After 6 min, a 10% aluminum nitrate solution (200 μL) was added and allowed to stand for another 6 min before 4% sodium hydroxide (2 mL) was added, followed by adjusting the volume to 5 mL with deionized water. The absorbance was measured after 15 min at 510 nm and compared to that of rutin standards (0–144 μmol/L). Flavonoid content of the sample was expressed as μmol of rutin eq./100 g DW.

### 3.6. Phenolic Compound Profiles in Tartary Buckwheat

The six tartary buckwheat samples were analyzed for their free, bound and total (free and bound) phenolic acid and flavonoid composition by HPLC (SPD-M10A VP Shimadzu, LC-8A pump, Japan) using a Phenomenex C18 column (250 mm × 4.6 mm) [30] and UV/VIS detector. The column temperature was 30 °C. Phenolic acids and flavonoid were detected at 300 nm and separated using a linear gradient elution program with a mobile phase containing solvent A (methanol/H_2_O/acetic acid, 65:34.5:0.5, v/v/v) and solvent B (H_2_O/acetic acid, 99.5:0.5, v/v). The gradient program was as follows: from 85% B to 70% B in 8 min, from 70% B to 65% B in 7 min, from 65% B to 25% B in 3 min, from 25% B to 85% B in 17 min. The flow rate used was set at 0.8 mL/min throughout the gradient. Identification and quantification was accomplished by comparing the retention time of peaks in the methanol solution to that of the standard compounds.

### 3.7. Determination of Antioxidant Activities

#### 3.7.1. DPPH Radical Scavenging Activity Assay

The method described by Abu Bakar *et al.* was used to assess the DPPH radical scavenging activity of tartary buckwheat phenolics [31]. One milliliter of the working sample solution or control was mixed with DPPH radical solution (1.0 mL). The mixture was shaken vigorously and left to stand at room temperature for 30 min in the dark. The mixture was measured spectrophotometrically at 517 nm. A standard curve was then prepared by plotting the percentage (%) of free radical scavenging activity of Trolox *versus* its concentration (0–65 μmol/L). The DPPH radical scavenging activity of the sample was expressed as μmol Trolox equivalents antioxidant capacity in 100 g DW of sample (μmol Trolox eq./100g DW).

#### 3.7.2. ABTS^●+^ Scavenging Activity Assay

Free radical scavenging capacity of the extracts was evaluated against ABTS^●+^ generated according to previously reported protocol [32]. ABTS^●+^ was generated by oxidizing 5 mM aqueous solution of ABTS (25 mL) with manganese dioxide (1.5 g) at ambient temperature for 30 min. The solution was then filtered to remove the existing manganese dioxide. The final reaction mixture contained 3.0 mL of ABTS^●+^ with an absorbance of 0.7 at 734 nm and 200 μL of the working sample solution or methanol for the control. Absorbance was measured at 734 nm after 1 min of reaction time. A standard curve was then prepared by plotting the percentage (%) of free radical scavenging activity of Trolox *versus* its concentration (0–29 μmol/L). The ABTS^●+^ scavenging activity of the sample was expressed as μmol Trolox eq./100g DW.

#### 3.7.3. β-carotene-linoleic Acid Assay

The antioxidant activity of extracts was determined by the method of Li and Zhou with slight modifications [33]. Two milliliters of 0.2 mg/mL β-carotene was dissolved in chloroform in round bottom flasks (100 mL) containing linoleic acid (45 mg) and Tween-40 emulsifier (350 mg). The chloroform was removed at 45 °C under vacuum. One hundred milliliters of aerated distilled water was added to the flask with vigorous shaking. Four milliliters were transferred into different test tubes containing 0.01 g/mL of free phenolics extracts or 0.1 g/mL of bound phenolics extracts (100 μL). Immediately after, the extracts were subjected to thermal auto-oxidant in a water bath at 50 °C for 60 min. Absorbance was measured after 60 min at 470 nm. A blank, without β-carotene, was prepared for background subtraction. The same procedure was repeated using methanol, serving as zero control. The zero control was measured at zero time before thermal oxidation and at 60 min after thermally oxidated. The AAC of the extracts was calculated using the following formula [28]:

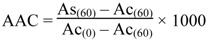

where As_(60)_ was the absorbance of the sample at t = 60 min, Ac_(60)_ the absorbance of the zero control (without sample ) at t = 60 min, and Ac_(0)_ the absorbance of the control at t = 0 min.

### 3.8. Statistical Analysis

Data from this study were reported as mean ± standard deviation (SD) for at least three replicates for each sample. Analysis of variance and least significant difference tests were conducted to identify difference among means using SPSS 18.0 software. Correlation analyses were performed using a Pearson correlation test. Statistical significance was declared at *P* < 0.05.

## 4. Conclusions

This study suggests that tartary buckwheat has the potential to provide health benefits because of its high phenolic content and antioxidant properties. *p*-Hydroxybenzoic, ferulic, protocatechuic, *p*-coumaric, gallic, caffeic, vanillic and syringic acids were detected, and *p*-hydroxybenzoic, ferulic and protocatechuic acids were the prominent phenolic acids in tartary buckwheat. The majority of phenolic compounds of tartary buckwheat were present in the free form and the distinction between free and bound phenolics helps to understand the potential benefit of tartary buckwheat consumption. Additionally, results from this study indicate that growing environment and the interaction between variety and environment may contribute more to individual antioxidant properties and phenolics of tartary buckwheat. Environmental parameter such as higher altitudes may also have an increasing effect on rutin and phenolic acids content of tartary buckwheat. These results also show the possibility of increasing the content of natural antioxidants by optimizing the growing conditions of a selected variety. Whereas the scope of the results from this study presents preliminary insights into how variety and growing environment may influence individual tartary buckwheat antioxidant properties and phenolics. More in-depth studies are required to better understand these complex relationships.
